# Hypoxia leads to significant changes in alternative splicing and elevated expression of CLK splice factor kinases in PC3 prostate cancer cells

**DOI:** 10.1186/s12885-018-4227-7

**Published:** 2018-04-02

**Authors:** Elizabeth Bowler, Sean Porazinski, Simon Uzor, Philippe Thibault, Mathieu Durand, Elvy Lapointe, Kasper M. A. Rouschop, John Hancock, Ian Wilson, Michael Ladomery

**Affiliations:** 1 Centre for Research in Biosciences, Faculty of Health and Applied Sciences, University of the West of England, Coldharbour Lane, Frenchay, Bristol, BS16 1QY UK; 2 Z8 Pavillon de Recherche Appliquée sur le Cancer (PRAC), Université de Sherbrooke, 3201 Jean-Mignault, Sherbrooke, Québec J1E 4K8 Canada; 3 Department of Radiation Oncology (Maastro Lab), GROW-School for Oncology and Developmental Biology, Maastricht University, Maastricht, The Netherlands

**Keywords:** Hypoxia, Alternative splicing, Prostate cancer, Apoptosis, Splice factors, Splice factor kinases, CLK1, CLK3, TG003

## Abstract

**Background:**

Mounting evidence suggests that one of the ways that cells adapt to hypoxia is through alternative splicing. The aim of this study was firstly to examine the effect of hypoxia on the alternative splicing of cancer associated genes using the prostate cancer cell line PC3 as a model. Secondly, the effect of hypoxia on the expression of several regulators of splicing was examined.

**Methods:**

PC3 cells were grown in 1% oxygen in a hypoxic chamber for 48 h, RNA extracted and sent for high throughput PCR analysis at the RNomics platform at the University of Sherbrooke, Canada. Genes whose exon inclusion rate PSI (ψ) changed significantly were identified, and their altered exon inclusion rates verified by RT-PCR in three cell lines. The expression of splice factors and splice factor kinases in response to hypoxia was examined by qPCR and western blotting. The splice factor kinase CLK1 was inhibited with the benzothiazole TG003.

**Results:**

In PC3 cells the exon inclusion rate PSI (ψ) was seen to change by > 25% in 12 cancer-associated genes; *MBP, APAF1, PUF60, SYNE2, CDC42BPA, FGFR10P, BTN2A2, UTRN, RAP1GDS1, PTPN13, TTC23* and *CASP9* (caspase 9). The expression of the splice factors SRSF1, SRSF2, SRSF3, SAM68, HuR, hnRNPA1, and of the splice factor kinases SRPK1 and CLK1 increased significantly in hypoxia. We also observed that the splice factor kinase CLK3, but not CLK2 and CLK4, was also induced in hypoxic DU145 prostate, HT29 colon and MCF7 breast cancer cell lines. Lastly, we show that the inhibition of CLK1 in PC3 cells with the benzothiazole TG003 increased expression of the anti-apoptotic isoform caspase 9b.

**Conclusions:**

Significant changes in alternative splicing of cancer associated genes occur in prostate cancer cells in hypoxic conditions. The expression of several splice factors and splice factor kinases increases during hypoxia, in particular the Cdc-like splice factor kinases CLK1 and CLK3. We suggest that in hypoxia the elevated expression of these regulators of splicing helps cells adapt through alternative splicing of key cancer-associated genes. We suggest that the CLK splice factor kinases could be targeted in cancers in which hypoxia contributes to resistance to therapy.

**Electronic supplementary material:**

The online version of this article (10.1186/s12885-018-4227-7) contains supplementary material, which is available to authorized users.

## Background

Hypoxia is defined as the state in which the availability or delivery of oxygen is insufficient to meet tissue demand [1]. It is a common feature of aggressive cancers, as angiogenesis cannot match the rate of tumour growth [2]. Insufficient angiogenesis results in poor delivery of nutrients, and this coupled with severe hypoxic stress can trigger apoptosis [3]. Remarkably, cancer cells are often able to survive the harsh conditions associated with hypoxia.

Hypoxic tumours are more difficult to treat. In radiotherapy, high-energy photons directed at the tumour site react with biological molecules to form DNA-damaging molecules, causing cancer cells to undergo apoptosis leading to shrinkage of the tumour [4]. Therefore, in hypoxic regions where there is a low level of oxygen molecules, radiotherapy is often not an effective form of treatment. In addition, tumour hypoxia can lead to resistance to chemotherapeutic drugs. A review published by Brown outlines three reasons for this [5]. Firstly, with diminished vasculature, drugs are unable to travel to hypoxic regions. Secondly, many chemotherapeutics target rapidly proliferating cells; however hypoxic cells tend to progress through the cell cycle much more slowly. Thirdly, hypoxia increases the expression of proteins involved in drug resistance, such as metallothionein-IIA (MT-IIA) involved in cisplatin resistance [6] and periostin involved in arsenic trioxide resistance [7]. A greater understanding of how tumour cells are able to adapt to hypoxia could help improve existing therapies or even suggest new treatment solutions.

Changes in alternative splicing of pre-mRNA may be one mechanism through which cells are able to adapt to hypoxic conditions. Pre-mRNA splicing involves the excision of introns and precise joining of exons to form the mature RNA [8]. 95% of pre-mRNAs are alternatively spliced in humans [9], giving rise to at least 100,000 distinct proteins [10] despite there only being an estimated 20,000 protein-coding genes. Splice isoforms encode proteins with distinct cellular functions. Hypoxia has been shown to have a significant effect on the alternative splicing of genes in normal endothelial cells [11, 12]; in cartilage endplate-derived stem cells [13]; in Hep3B liver [14] and in MCF7 breast cancer cell lines [15]. Hypoxia reduces expression of MAX (MYC-associated factor X) in endothelial cells through increased unproductive splicing [16]. The splicing of the *SMN2* gene is also modified by hypoxia in the context of Spinal Muscular Atrophy [17]. While several studies utilised exon arrays to study changes in exonic splicing during hypoxia, a recent study conducted in MCF7 breast cancer cells utilised whole transcriptome RNA-seq identifying intron retention as the most commonly altered alternative splicing event [15].

Given these notable changes in alternative splicing in hypoxia, the question is which regulators of splicing are involved? The aim of the present study is firstly to examine the effect of hypoxia on alternative splicing in a prostate cancer cell line model, and secondly to examine the expression of several splice factors and splice factor in response to hypoxia.

## Methods

### Cell lines, hypoxia treatments and CLK1 inhibition

The prostate cancer cell lines, PC3 (Sigma-Aldrich, 90112714), VCaP (Sigma-Aldrich, 06020201) and DU145 (ATCC, HTB-81), the normal prostate epithelial PNT2 cell line (ECACC, 95012613), the colorectal cancer cell line HT29 (Sigma-Aldrich, 91072201), and the breast cancer cell line MCF7 (Sigma-Aldrich, 86012803) were cultured in high glucose Dulbecco’s modified Eagle’s medium (DMEM), supplemented with 10% fetal bovine serum (FBS) and 1% L-glutamine-penicillin-streptomycin and grown at 37 °C with 5% CO_2_. Hypoxia was attained in a modular incubator chamber (MIC-101; Billups-Rothenberg, USA) using a gas mixture containing; 1% O_2_ / 5% CO_2_ / 94% N_2_ (BOC, UK). The chamber was flooded with the hypoxic gas mixture for 2 min and then sealed and stored in an incubator for 48 h at 37 °C in 5% CO_2_. The normoxic control was stored in the same incubator for the same amount of time. CLK1 was inhibited with the benzothiazole TG003 (Sigma-Aldrich) at 10–50 μM; the TG003 stock was dissolved in dimethylsulphoxide (DMSO).

### RNA extraction

RNA was extracted using Tri reagent (Sigma-Aldrich) as per manufacturer’s instructions with the following modifications; the first three centrifugations (homogenate, phase separation and RNA isolation) were conducted at 10,000 g, phase separation centrifugation was for 30 min; and 0.1 ml chloroform was used for phase separation. RNA pellets were washed with 0.5 ml 70% ethanol and centrifuged at 3500 g, and the wash step was repeated twice. RNA pellets were resuspended in 30 μl nuclease-free H_2_O (Promega). Genomic DNA was removed using the Precision DNase kit (Primerdesign, UK). Total RNA concentration was determined using a Nanodrop spectrophotometer (ThermoFisher Scientific, Delaware).

### cDNA synthesis and standard PCR

cDNA was synthesised with the GoScript reverse transcription system (Promega, UK). Total RNA was reverse transcribed using a 1:1 mixture of random primers and oligo(dT). The GoTaq hot start polymerase kit (Promega, UK) was used to perform standard PCR. PCR conditions were: initial denaturation at 94 °C for 2 min; then 35–40 cycles: 94 °C for 30 s; 58 °C for 30 s; and 72 °C for 30 s followed by a final extension at 72 °C for 5 min. Primer sequences used in standard PCR are shown in Additional file 1: Table S1. PCR products were run on 2% (*w*/*v*) agarose gels for 1 h at 95 V and analysed using FluorChem Q software, Alpha Innotech MultiImage III. Optical density peak values were generated with ImageJ software. Excel was used to calculate the percentage splicing index (PSI/ψ) where ψ = exon inclusion/ exon inclusion + exon skipping band intensities.

### High-throughput PCR

High-throughput PCR was performed at the RNOmics platform at the University of Sherbrooke, Canada. RNA integrity was determined using an Agilent 2100 Bioanalyzer (Agilent Technologies). 2.2 μg total RNA were reverse transcribed with Transcriptor reverse transcriptase, random hexamers, dNTPs (Roche Diagnostics), and 10 units of RNAse OUT (Invitrogen) in 20 μl. All primers were resuspended in 20–100 μM stock solutions in Tris-EDTA buffer (IDT) and diluted as primer pairs to 1.2 μM in RNase and DNase-free water (IDT). RT-PCR reactions were performed using 10 ng cDNA as template in a 10 μL final volume containing 0.2 mmol/L dNTP, 1.5 mmol/L MgCl_2_, 0.6 μmol/L each primer, and 0.2 units of Platinum Taq DNA polymerase (Invitrogen). After an initial denaturation at 95 °C for 2 min, 35 PCR cycles were performed; 94 °C 30 s, 55 °C 30 s, and 72 °C 60 s, followed by a final extension step at 72 °C for 2 min. PCR reactions were carried out on the GeneAmp PCR System 9700 (ABI), and the amplicons were examined by automated microcapillary electrophoresis using Caliper LC-90 instruments (Caliper LifeSciences). The percent spliced index (PSI, ψ) was calculated as the ratio of the band intensity of the exon inclusion amplicon divided by the sum of both inclusion and exclusion amplicon band intensities, measured using ImageJ software. The selection of cassette exons was based on genes from the NCBI RefSeq database associated with cancer and apoptosis pathways, all of which suitable for PSI analysis [18, 19].

### qRT-PCR

qRT-PCR was performed in 96-well plates as per the protocol by Primerdesign (UK), using SensiFAST Sybr Hi-Rox kit (Bioline, UK) mastermix. Three technical repeats were conducted per experimental sample, and nuclease free water was used as a negative control. qRT-PCR plates were run on an ABI 7300 qRT-PCR thermal cycler (Applied Biosciences). PCR conditions were as follows; initially 95 °C for 10 min; then 95 °C for 15 s; 60 °C for 1 min for 40 cycles. Melting curve conditions were 95 °C for 15 s; 60 °C for 1 min; 95 °C for 15 s. Primer sequences used in qRT-PCR are shown in Additional file 2: Table S2. CT values were normalised to the *UBC* and *RPL13A* housekeeping genes. All results were calculated using Excel software (Microsoft, USA).

### Protein extraction and quantification

For the extraction of protein, cells were washed with phosphate buffered saline (PBS) before the addition RIPA lysis buffer (Sigma, UK) and phosphatase inhibitor tablets (Complete Mini EDTA-free PI tablets, Roche Diagnostics, UK). Cell lysates were collected in 1.5 ml Eppendorf tubes and kept on ice. The Pierce BCA assay (Thermo Fisher Scientific, USA) was used to measure protein concentration.

### Western blotting

Protein samples were mixed 1:1 with 2× Laemmli buffer (Sigma, UK) and incubated at 100 °C for 5 min before being chilled on ice. Protein samples of equal amounts were subjected to SDS-PAGE and transferred to PDVF membranes. Membranes were incubated at 4 °C overnight with one of the following antibodies; anti-CA IX (M75 monoclonal antibody, Novus Biologicals); anti-SRSF1 (sc-33652, SantaCruz Biotechnology, UK); anti-CLK1 (R1471–1 s, Abiocode, USA); anti-phospho SR(1H4) (sc-13509, SantaCruz Biotechnology, UK); anti-β-actin (ab8226, Abcam, UK). Horse anti-mouse IgG HRP-linked antibody (7076S, Cell Signalling, UK) was used to detect all antibodies apart from anti-CLK1, which was detected by goat anti-rabbit IgG HRP-linked antibody (7074, Cell Signalling, UK). Laminata Forta Western HRP substrate (Millipore, UK) was used to image protein bands.

### Analysis of promoter sequences

The promoter sequences of *SRSF1, SRSF2, SRSF3, SAM68, HuR, hnRNP A1, CLK1* and *SRPK1* splice factors and splice factor kinases were examined for the presence of HIF response element (HRE) sequences (5’-RCGTG-3′) using the Eukaryotic Promoter Database (http://epd.vital-it.ch/). Parameters were set from − 1000 to + 100 of the transcription start site (TSS), as HIF-1α mostly binds close to the TSS [20].

### Statistical analysis

All data obtained for the standard and qRT-PCR experiments was subjected to Shapiro-Wilks and Bartlett’s test in order to deduce the distribution and variance of the data, respectively. For the standard PCR, data that was found to be normally distributed and homoschedastic was then subjected to a student’s T-test in order to examine the statistical significance of the data. Results found not to be normally distributed and/or heteroschedastic were subjected to a Mann Whitney U statistical test of significance. A Mann Whitney U non-parametric test was used for test for statistical significance for all the real-time PCR data. For all results obtained from the standard PCR and real-time PCR experiments, the means were calculated from the results and plotted on graphs, and error bars were added using the 95% confidence interval.

## Results

### High-throughput PCR analysis of alternative splicing in hypoxic PC3 cells

**Fig. 1:**
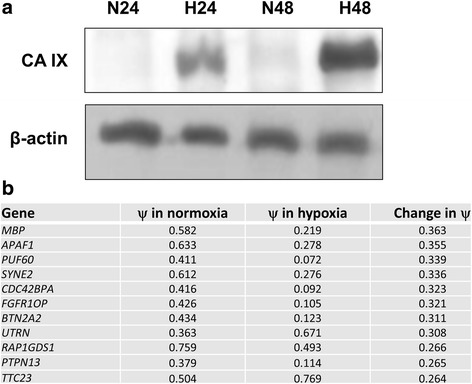
Exposure of PC3 cells to hypoxia (1% oxygen) results in changes in alternative splicing of cancer associated genes. PC3 prostate cancer cells were exposed to 1% oxygen or normoxic conditions for up to 48 h. **a** Protein samples were extracted at 24 and 48 h time points and western blotted for CA IX (carbonic anhydrase 9), a hypoxia marker; N refers to normoxia and H to hypoxia. β-actin was used as a loading control. **b** RNA samples were extracted at 48 h and sent to the RNomics platform at the University of Sherbrooke, Canada, for high-throughput PCR analysis of the rate of inclusion of 600 cancer-associated cassette exons. Genes whose ψ indexes (proportion of exon included) changed by > 25% are listed

It is clear that cells can adapt to hypoxic conditions by altering alternative splicing programmes [11–17]; however the effect of hypoxia on alternative splicing in prostate cancer cells has not been examined. We utilised a hypoxic chamber, perfused with an air mixture containing 1% oxygen, and exposed PC3 cells (androgen independent, established from a grade 4 prostatic adenocarcinoma) to these conditions for up to 48 h. To confirm the establishment of a hypoxic environment we measured the hypoxia marker carbonic anhydrase IX (CA IX). CA IX is highly induced in hypoxia as it facilitates the adaptation of cells to a severely altered pH environment [21]. Consistent with hypoxic conditions a significant induction of CA IX was observed (Fig. 1a). RNA was extracted from PC3 cells 48 h after seeding, one set of cells growing in normoxia and another in 1% oxygen for 48 h. The RNA was sent for analysis at the RNOmics platform at the University of Sherbrooke, Canada. The RNOmics platform utilises a high-throughput reverse transcription-PCR platform to analyse cassette exon events associated with 600 cancer-associated genes; the approach is called LISA (Layered and Integrated system for Splicing Annotation) [18, 19]. Cassette exon inclusion is measured as the value PSI (ψ) where 1 = complete inclusion, and 0 complete exon skipping. Out of the LISA data set we found that 11 genes showed a change in ψ value greater than 25% compared to normoxic values (Fig. 1b; *MBP, APAF1, PUF60, SYNE2, CDC42BPA, FGFR10P, BTN2A2, UTRN, RAP1GDS1, PTPN13,* and *TTC23*). We note that several of the genes are involved in the regulation of cytoskeletal architecture. The properties of the alternative splice isoforms are summarised in Table 1.

**Table 1:** List of genes whose ψ value change by over 25% in hypoxia

Gene name	Gene function	Function of splice isoform	Change in splice isoform expression after hypoxia
*APAFl*	Required for the formation of the apoptosome in apoptosis [32]	Exon 17a codes for a WDR domain required for the formation of the apoptosome [33]	Exon 17a skipping is favoured in hypoxia
*BTN2A2*	Involved in immune tolerance in cancer	Exon 5 skipping forms a truncated protein that negatively regulates the full length protein involved in immune tolerance [39]	Increased exon inclusion, which promotes immune tolerance
*CDC42BPA*	Reorganisation of the cytoskeleton, formation of filopodia and assignment of cellular polarity. Implicated in cancer cell motility and invasion	Unknown function	Decrease in exon inclusion in hypoxia.
*FGFR10P*	Ciliogenesis, cellular motility, cell growth and cell cycle progression	Unknown function	Exon skipping favoured in hypoxia
*MBP*	Formation of the myelin sheath. Elevated in breast cancer and lung cancer patients with brain metastasis	Unknown function.	Exon skipping favoured in hypoxia.
*PTPN13*	Competing roles as a tumour suppressor and oncogene	Unknown function.	Decrease in exon inclusion in hypoxia
*PUF60*	Modulates alternative splicing through recognition of 3′ splice sites. Regulates *c-myc* transcription	Exon 5 skipping results in expression of an isoform known as FIR, a *c-myc* repressor [40, 41]	Expression of anti-oncogenic FIR isoform favoured in hypoxia.
*RAP1GDS1*	Activates multiple small GTPases in the Rho and Ras families	Exon 5 skipping produces SmgGDS-558 which plays a greater role in proliferation and NFkB activity than the full-length SmgGDS-607 splice variant [42]).	Hypoxia favours the smgGDS-558 isoform.
*SYNE2*	Influences the shape and migration properties of cells	Exon 107 encodes a domain required for scaffolding for protein-protein interactions [43].	Exon skipping favoured in hypoxia
*TTC23*	Unknown function but expression linked to cervical, bladder and prostate cancers.	Unknown function	Exon inclusion favoured in hypoxia.
*UTRN*	Maintenance of the cytoskeleton	Unknown function	Exon skipping increased in hypoxia.

### Verification of high-throughput PCR results

**Fig. 2:**
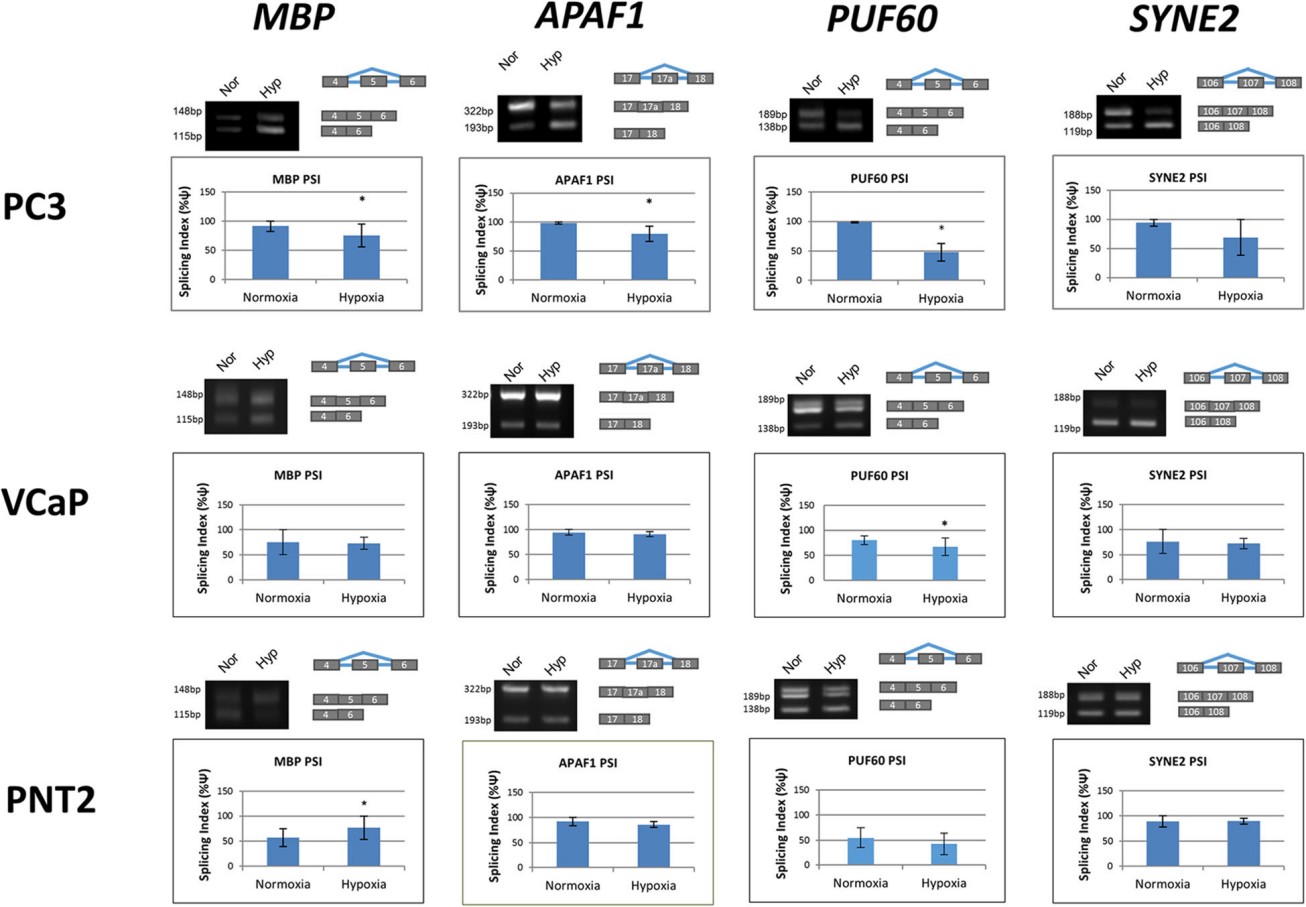
Verification of high-throughput PCR results. RNA was extracted from PC3 (*N* = 5) and VCaP (*N* = 3) prostate cancer, and PNT2 (*N* = 3) normal prostate epithelium cell lines after 48 h in normoxia or 1% in hypoxia. For each gene a representative PCR is shown, together with the cassette exon involved. The average splicing index ψ (proportion of exon included) is shown graphically. The genes are*MBP, APAF1, PUF60 and SYNE2*

**Fig. 3:**
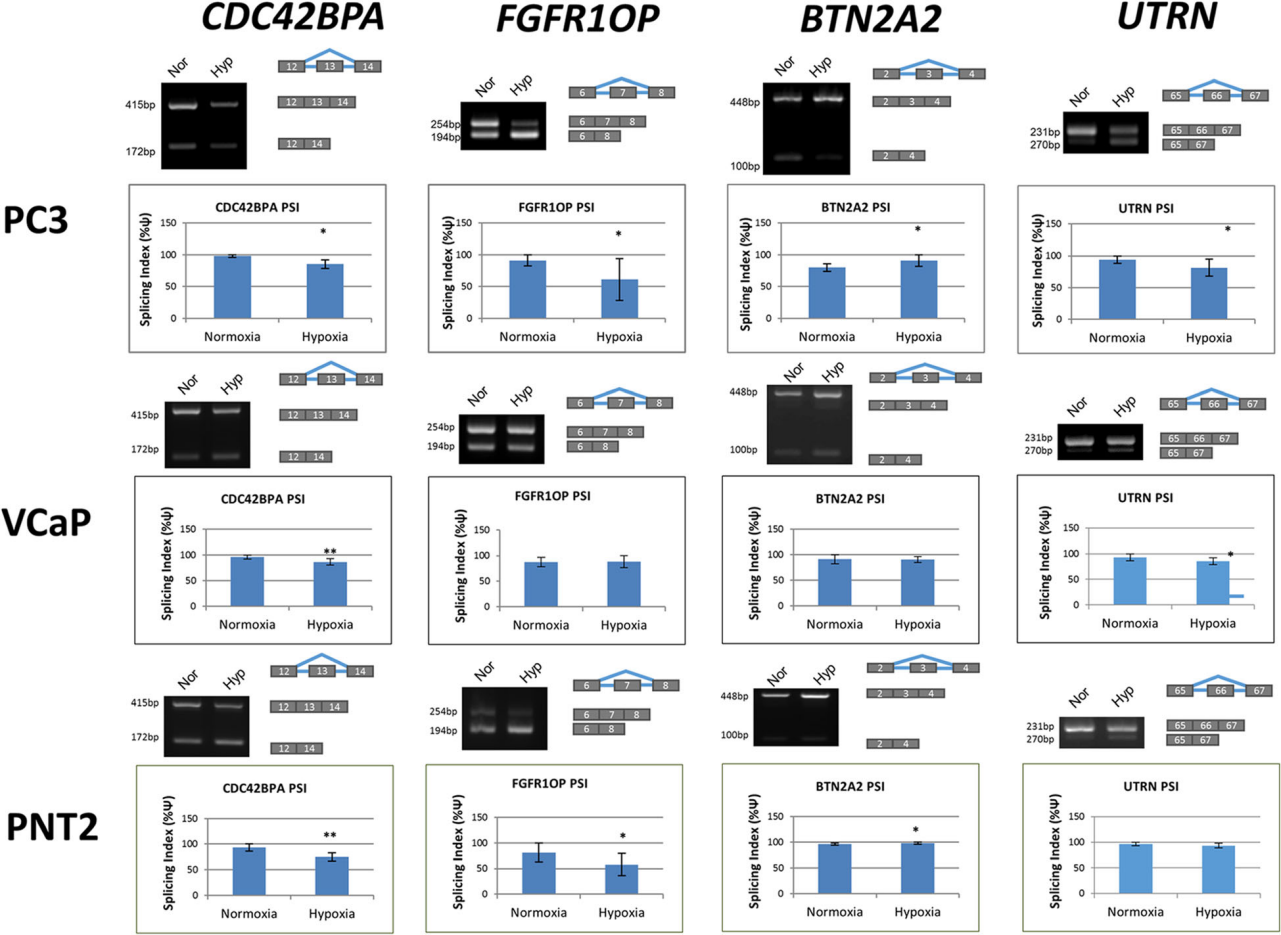
Verification of high-throughput PCR results. RNA was extracted from PC3 (*N* = 5) and VCaP (*N* = 3) prostate cancer, and PNT2 (*N* = 3) normal prostate epithelium cell lines after 48 h in normoxia or 1% in hypoxia. For each gene a representative PCR is shown, together with the cassette exon involved. The average splicing index ψ (proportion of exon included) is shown graphically. The genes are *CDC42BPA, FGFR10P, BTN2A2, UTRN*

**Fig. 4:**
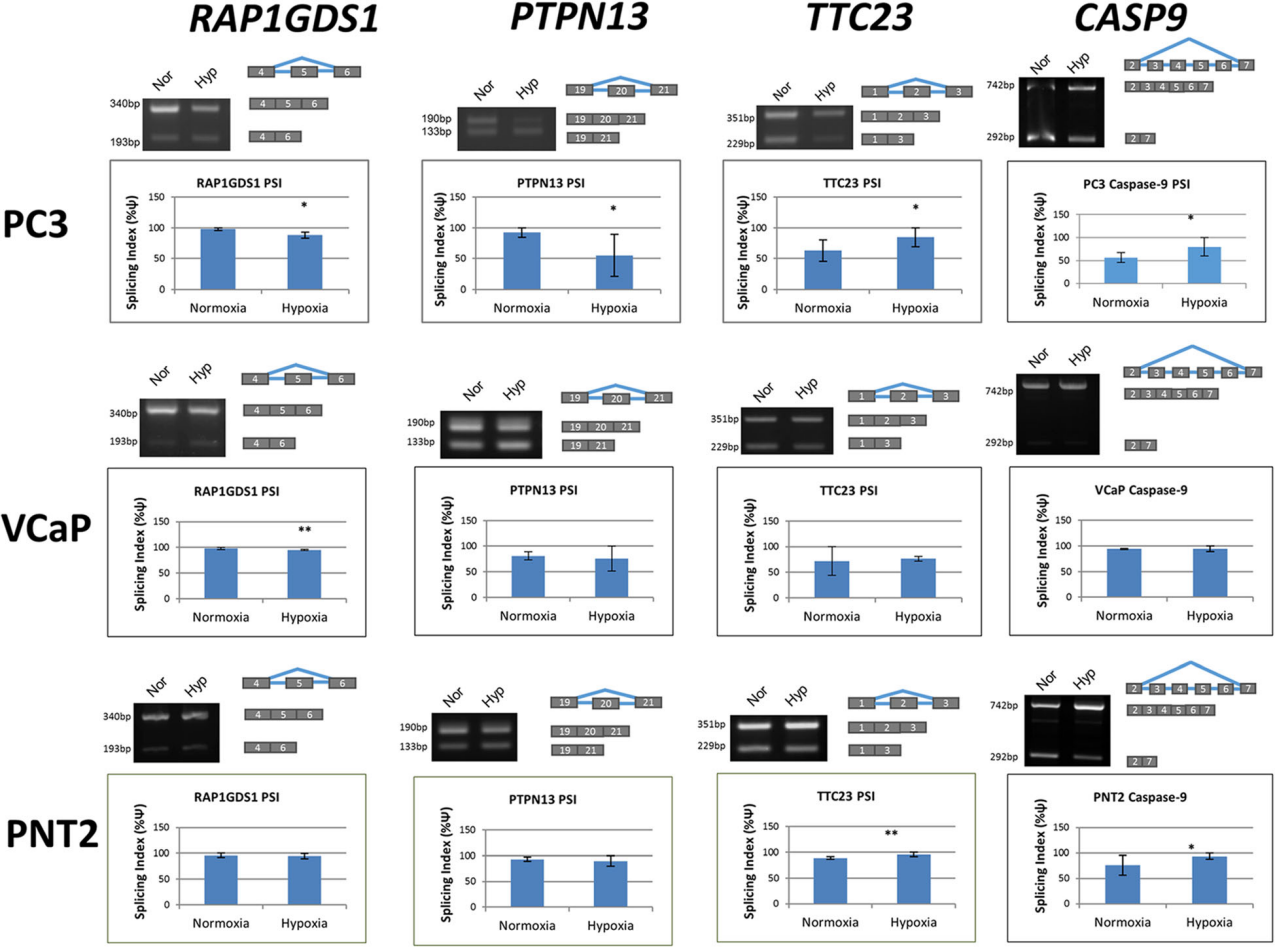
Verification of high-throughput PCR results. RNA was extracted from PC3 (*N* = 5) and VCaP (*N* = 3) prostate cancer, and PNT2 (*N* = 3) normal prostate epithelium cell lines after 48 h in normoxia or 1% in hypoxia. For each gene a representative PCR is shown, together with the cassette exon involved. The average splicing index ψ (proportion of exon included) is shown graphically. The genes are *RAP1GDS1, PTPN13, TTC23* and *CASP9*

In order to validate the LISA results the experiment was repeated in three cell lines. In each case an equivalent number of PC3 and DU145 prostate cancer cells and the normal epithelial cell line PNT2 were exposed to 1% oxygen for 48 h. CA IX levels were elevated in each repeat experiment (data not shown). RNA was extracted and cDNA synthesized, and PCR performed using the same primers used in the RNOmics platform high-throughput PCR assay (Additional file 1: Table S1). To the set of 11 genes from the LISA analysis, we added *CASP9 (caspase-9)*, an important apoptosis marker (not present in the Sherbrooke LISA gene set). *CASP9* expresses several splice isoforms. Caspase 9b arises from skipping of exons 3–6, resulting in a truncated, anti-apoptotic isoform, whereas caspase 9a includes exons 3–6 encoding the functional pro-apoptotic isoform. Changes in ψ were statistically consistent in PC3 cells, confirming the findings of the high-throughput PCR. Quantification of PCR bands using Image J was performed for each of the repeats and a representative PCR gel is shown in each case (Figs. 2, 3, and 4).

### Expression of splice factor kinases in hypoxic PC3 cells

**Fig. 5:**
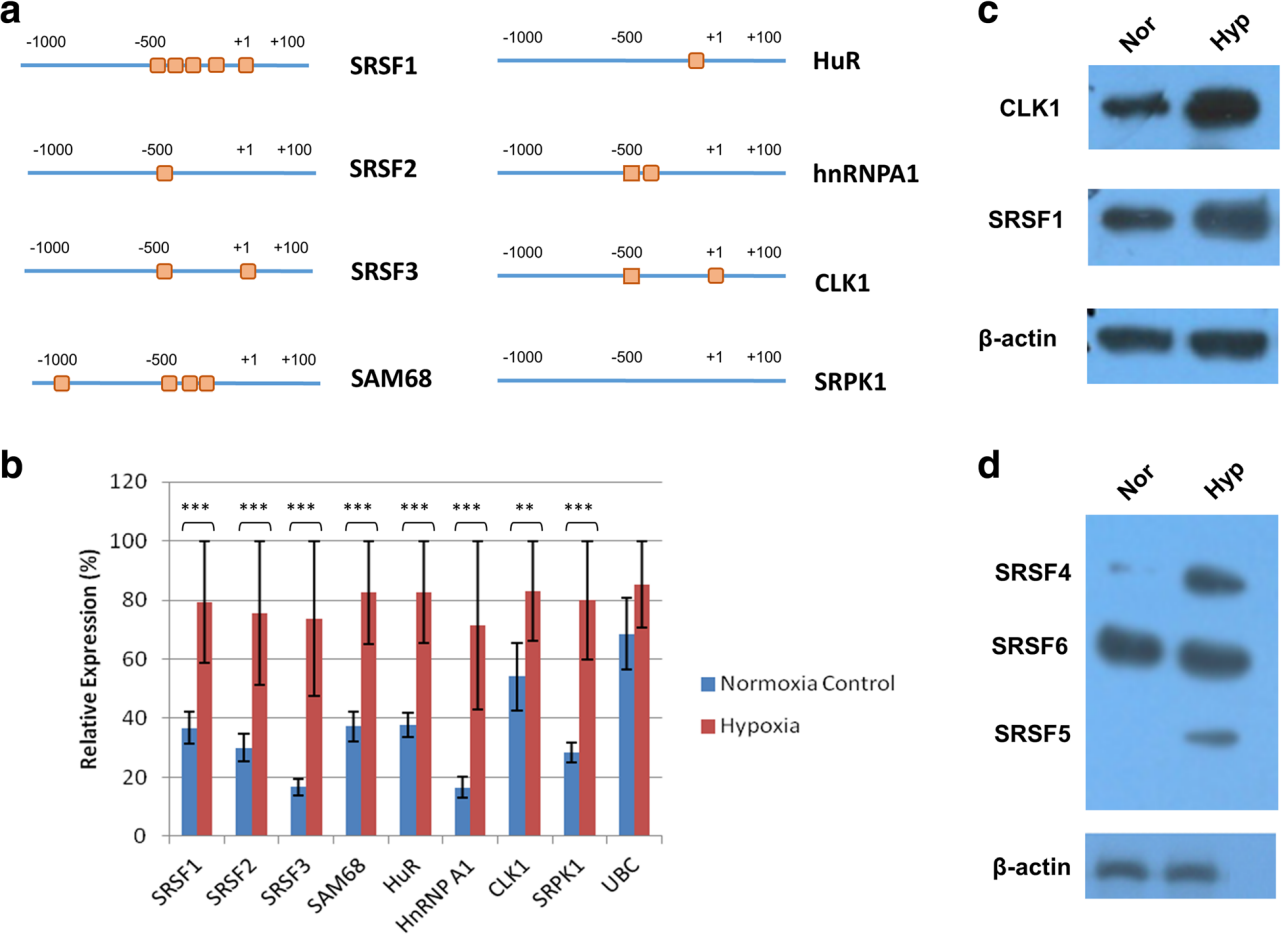
Changes in the expression of splice factors and splice factor kinases in response to hypoxia. **a** This panel illustrates the presence of potential hypoxia inducible factor (HIF) binding sites in the promoters of six splice factor (SRSF1, SRSF2, SRSF3, SAM68, HuR, hnRNPA1) and two splice factor kinase genes (CLK1, SRPK1), orange boxes. **b** Quantitative PCR measurement of splice factor and splice factor kinase expression, PC3 cells in normoxia or hypoxia for 48 h. *T*he housekeeping gene was *UBC*. **c** Western blot confirming increased levels of CLK1 and its substrate SRSF1 in hypoxia (PC3 cells, 48 h at 1% oxygen). β-actin is the loading control. **d** Levels of phosphorylated SR proteins measured with the mAb104 antibody that preferentially binds phosphorylated SR proteins

Changes in alternative splicing in hypoxia are likely to arise from changes in the expression and activity of splice factors and of splice factor kinases that modulate their function. We began by selecting a set of splice factors that have been shown to be associated with abnormal splicing patterns in cancer. These include three SR (serine- arginine rich) proteins SRSF1 (a proto-oncogene [22]), SRSF2 and SRSF3; the splice factor SAM68 (involved in signal transduction), HuR and hnRNPA1; and the splice factor kinases CLK1 and SRPK1. Several publications have identified a core hypoxia inducible factor (HIF) response element that is conserved from animals to zebrafish (5’-RCGTG-3′). Therefore, we examined their promoter sequences using the eukaryotic promoter database with parameters set from − 1000 to + 100 of the transcription start site, and noticed the presence of 1–5 HIF binding sites (Fig. 5a). HIF binding sites were present in all gene promoters except for the splice factor kinase SRPK1 (Fig. 5a). Next we examined their expression in hypoxia using qRT-PCR. The expression of all splice factor and splice factor kinases appeared to increase in hypoxia (Fig. 5b). Given that CLK1 has previously been shown to be HIF-inducible [23] we confirmed its increased expression, along with its substrate the splice factor SRSF1 by western blot (Fig. 5c). We also used a monoclonal antibody, mAb104, that specifically recognises phosphorylated SR proteins [24] and noticed elevated phosphorylation of SR proteins in hypoxia, consistent with an increased activity of splice factor kinases (Fig. 5d).

### Induction of CLK family expression in hypoxia

**Fig. 6:**
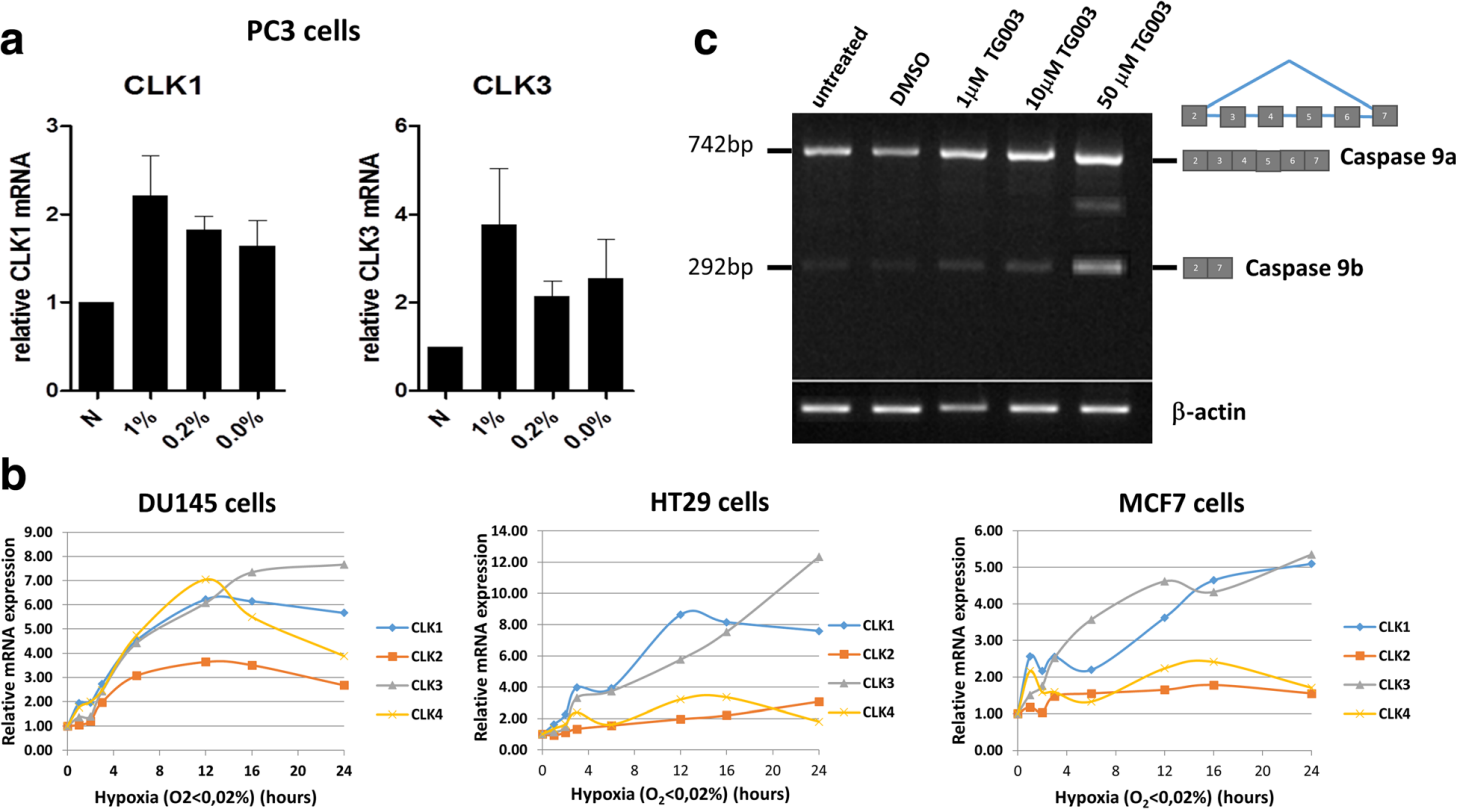
Changes in the expression of the CLK family of splice factor kinases in response to hypoxia and the effect of CLK1 inhibition on caspase 9 splicing. **a** PC3 cells were exposed for 16 h to three different hypoxic conditions; 1.0%, 0.2% and 0.0% oxygen and N (normoxia). The relative expression of CLK1 and of its homologue CLK3 are shown, normalised to the housekeeping gene *RPL31A*. **b** PC3 cells were treated for 48 h with the CLK1 inhibitor TG003; the effect on alternative splicing of *CASP9* is shown. **c** Microarray time-course showing the expression of four CLKs over 24 h in 0% hypoxia in DU145 (prostate), HT29 (colon) and MCF7 (breast) cancer cell lines

We examined the expression of the CLK (CDC-like) splice factor kinase CLK1 in more detail. The CLK family comprises four genes in humans, termed *CLK1–4*. Among these, CLK1 and CLK4 are very similar in amino-acid sequence. CLK1, CLK2 and CLK4 are ubiquitously expressed, whereas CLK3 is most expressed in testis [25]. Among the CLKs, CLK1 is the most highly studied, and its interaction with its substrate SRSF1 is well understood [26]. Firstly we looked at the induction of CLK1, and for comparison CLK3 in PC3 cells in three different hypoxic conditions, 1.0%, 0.2% and 0.0% oxygen. We observed an induction of CLK1 in all three hypoxic conditions after 16 h (Fig. 6a); unexpectedly, we also observed that CLK3 was induced. For comparison, Affymetrix microarray experiments were performed on DU145 prostate, HT29 colorectal and MCF7 breast cancer cell lines exposed to 0.0% hypoxia [27]. We wanted to determine whether or not CLK induction during hypoxia is specific to PC3 prostate cancer cells. We observed strong induction of CLK1 and CLK3 in all three cell lines, but not CLK2 and CLK4 (Fig. 6b). Finally, we inhibited CLK1 activity in PC3 cells using the benzothiazole inhibitor TG003 [28] in order to determine the effect on *CASP9* alternative splicing. We found that the expression of caspase 9b, clearly reduced in hypoxia (Fig. 4c), increased following CLK1 inhibition (Fig. 6c).

## Discussion

Recently there has been interest in examining hypoxia-driven changes in alternative splicing. A genome-wide study was conducted in hypoxic endothelial cells using the Affymetrix human exon 1.0 array [12]. Alterations were found in 294 genes including nine alternative splicing events that were not previously related to hypoxia. The changes involved to a large extent genes involved in angiogenesis, glucose metabolism, cell cycle, DNA repair, and cytoskeletal remodelling. In the DNA damage response, hypoxia drives the alternative splicing of genes towards noncoding isoforms through increased intron retention [29]. In a similar vein, in endothelial cells hypoxia promotes the expression of a splice isoform of MAX (Myc associated factor X) that is degraded by nonsense-mediated decay, and another splice isoform that encodes an unstable protein; the net effect is less active MAX protein is produced [16]. Investigations using the Hep3B hepatocarcinoma cell line revealed the hypoxia altered alternative splicing patterns of HIF and non-HIF genes including *CA IX, RAP1GDS1* and *MBP* [14]; *MBP* was also identified in our high throughput PCR screen (Fig. 1b). In MCF7 breast cancer cells hypoxia was found to affect the alternative splicing of genes that are involved in a number of cancer-driving processes including angiogenesis, cell growth and apoptosis, suggesting that hypoxia plays a role in cancer progression [15]. Furthermore, the latter study also confirmed intron retention as the most common type of splicing event to be altered in hypoxia. Typically, intron retention can result in the use of a premature stop codon, which in turn can trigger nonsense mediated decay (NMD) or produce a truncated protein. Thus there are a number of clearly documented cases of cells adapting to hypoxia by changing alternative splicing of key genes [11–17]. In the present study we can confirm that changes in alternative splicing of cancer genes also occur in response to hypoxia in the widely used prostate cancer cell lines PC3 and VCaP, and in the normal epithelial cell line PNT2.

Hypoxic tumour cells can become resistant to apoptosis by expressing apoptosis inhibitors such as IAP-2 [30]. There are several components of the apoptosis machinery and genes express pro- or anti-apoptotic splice isoforms (*BCL2L1, MCL1, CASP9* and *APAF1* are notable examples). A recent study shows that the pharmacological inhibition of APAF1 allows cells in which apoptosis is induced by hypoxia to recover [31–33]. We examined the alternative splicing of *CASP9*, and noted that in hypoxia expression of the pro-apoptotic full length caspase 9a isoform increased significantly in PC3 and PNT2 cells (Fig. 4c). We suggest that changes in alternative splicing in hypoxia result in a different balance of splice isoforms that contribute to the induction of apoptosis.

We examined the promoters of a representative set of splice factors and splice factor kinases, and found putative HIF consensus binding sites in 7/8 (the exception being SRPK1). The expression of all eight splice factors and kinases appeared to increase in hypoxia, suggesting a general activation of the machinery of alternative splicing in conditions of severe hypoxic stress. We turned our attention to the CDC-2 like (CLK) family of splice factor kinases, because recent literature has identified an increase in expression of CLK1 in response to hypoxia [23, 34]. We confirmed the induction of CLK1, not just in PC3 cells, but also in another prostate cancer cell line, DU145, and in HT29 colorectal and MCF7 breast cancer cell lines (Fig. 6a and b). We observed the parallel induction of CLK1 and CLK3 in the four cell lines. CLK3 is best known for its expression in normal testis [25]; however we suggest that along with CLK1, CLK3 might contribute to adaptation to hypoxia in cancer cells.

To address the functional significance of CLK1 induction in the context of alternative splicing in hypoxia, we treated PC3 cells with the CLK1 inhibitor, the benzothiazole TG003 [28]. Inhibition of CLK1 with up to 50 μM TG003 increased the expression of caspase 9b, the anti-apoptotic isoform in which exons 3–6 are skipped (Fig. 6c). There is interest in the potential of targeting CLK1 in the context of the influenza [35] and HIV-1 viruses [36] and in cancer cells [37, 38]. The fact that CLK kinases appear to help cancer cells adapt to hypoxia suggests that they could be useful therapeutic targets.

## Conclusions

This study demonstrates that there are significant changes in exon inclusion rates in a range of cancer-associated genes in PC3 prostate cancer cells exposed to 1% oxygen hypoxic conditions. The expression of splice factors and splice factor kinases also increases significantly in hypoxia; in particular we observed a consistent induction of CLK1 and CLK3 in four independent cell lines. We suggest that targeting CLK kinases may provide benefit in the treatment of cancers in which tumour hypoxia contributes to resistance to therapy.

## Additional file

Additional file 1: **Table S1.** Forward (F) and reverse (R) primer sequences for all human genes amplified using standard PCR. The target sites of the primers, including the exonic locations, are indicated. (DOCX 13 kb)

Additional file 2: **Table S2.** Forward and reverse primer sequences for all human genes amplified using qRT-PCR. Details of the primers using in qRT-PCR analysis. (DOCX 13 kb)

## Abbreviations

CLK: cdc-like kinase
HIF: Hypoxia inducible factor
SR: Serine-arginine rich
