# Supplementary material for: Hypoxia leads to significant changes in alternative splicing and elevated expression of CLK splice factor kinases in PC3 prostate cancer cells
Source: BMC Cancer. 2018 Apr 2;18:355. doi: 10.1186/s12885-018-4227-7 (PMC5879922; doi:10.1186/s12885-018-4227-7)
Supplement: Supplementary file 1 — Table S1. Forward (F) and reverse (R) primer sequences for all human genes amplified using standard PCR. The target sites of the primers, including the exonic locations, are indicated. (DOCX 13 kb) [file 12885_2018_4227_MOESM1_ESM.docx]

| **Gene Name** | **Primer Sequences** | **Target Site** |
| --- | --- | --- |
| *CA IX*  NM_001216 | **F:** CTCTGACTACACCGCCCTGTG  **R:** GCGGTAGCTCACACCCCCTTT | 1031-1051 (Exon 7)  1392-1372 (Exon 10) |
| *β-actin*  NM_001101 | **F:** CCTGGCACCCAGCACAAT  **R:** GCCGATCCACACGGAGTACT | 1041-1058 (Exon 5)  1110-1091 (Exon 6) |
| *BTN2A2*  NM_006995 | **F:** CCTGCTCCTCCTCCTTCTCAGC  **R:** CAGCCAGATGCTCCCATCCTC | 165-186 (Exon 2)  612-592 (Exon 4) |
| *INSR*  NM­_000208 | **F:** TGAGGATTACCTGCACAACG  **R:** GCTGGTCGAGGAAGTGTTG | 2607-2626 (Exon 10)  2772-2754 (Exon 12) |
| *UTRN*  NM­_007124 | **F:** CAAACACCCTCGACTTGGTT  **R:** TGGCAATACTGCTGGATGAG | 9524-9543 (Exon 65)  9793-9774 (Exon 69) |
| *FGFR1OP*  *­*NM­_007045 | **F:** CTGTGGGTGGACCCTTATTATTAG  **R:** GACACTTGTATCACTCTGATTGGC | 461-484 (Exon 5)  714-691 (Exon 8) |
| *CDC42BPA*  NM_003607 | **F:** GCTAATGCTGTGAGGCAAGAAC  **R:** GCTCACTCTGTTCACGTAGCTT | 2489-2510 (Exon 12)  2903-2882 (Exon 14) |
| *SYNE2*  NM_182914 | **F:** CTCACGAAGAGGACGAGGAG  **R:** TTGCTTGTAGTGATGCTCGG | 19519-19538 (Exon 106)  19706-19687 (Exon 108) |
| *APAF1F1*  NM_181861 | **F:** CTTTGGGATGCGACATCAGCAA  **R:** CCACCTTTGAACGTGAGTCTGT | 2882-2903 (Exon 17)  3074-3053 (Exon 18) |
| *APAF1F8*  NM_181861 | **F:** GTGAAGTGTTGTTCGTGGTCTG  **R:** CATCACACCATGAACCCAAC | 2978-2999 (Exon 17)  3244-3225 (Exon 18) |
| *PUF60*  NM_078480 | **F:** GCCAAGAAGTACGCCATGG  **R:** GTAGACGCGGCACATGATG | 291-309 (Exon 4)  479-461 (Exon 6) |
| *MBP*  NM_001025081 | **F:** CTCGCACACCACCCCCGTC  **R:** TCGACTCCCTTGAATCCCTTGT | 459-477 (Exon 4)  606-585 (Exon 6) |
| *LHX6*  NM_014368 | **F:** TCCGACGACATCCACTACAC  **R:** TGGTGGGTTCTGGTTCTCAG | 1051-1070 (Exon 8)  1441-1422 (Exon 10) |
| *PTPN13*  NM_080685 | **F:** GACTCCTCATCCATTGAAGACC  **R:** CCAAGCCATACTTTGCATCTTT | 3604-3625 (Exon 19)  3793-3772 (Exon 21) |
| *RAP1GDS1*  NM_001100426 | **F:** TCCATGTGTGGATGCTGGATTG  **R:** TGCATTTTGGCAGTGGATGC | 442-463 (Exon 4)  781-762 (Exon 6) |
| *TTC23*  NM_001288615 | **F:** AAGGAGTGATTGAGTGTCAAAGGA  **R:** CGTGGGTTCAGCTTTCTAGGTC | 304-327 (Exon 1)  654-633 (Exon 3) |
| *CASP9*  NM_001229 | **F:** GCTCTTCCTTTGTTCATCTC  **R:** CATCTGGCTCGGGGTTACTGC | 450-470 (Exon 2)  1191-1171 (Exon 7) |
