# Supplementary material for: Hypoxia leads to significant changes in alternative splicing and elevated expression of CLK splice factor kinases in PC3 prostate cancer cells
Source: BMC Cancer. 2018 Apr 2;18:355. doi: 10.1186/s12885-018-4227-7 (PMC5879922; doi:10.1186/s12885-018-4227-7)
Supplement: Supplementary file 2 — Table S2. Forward and reverse primer sequences for all human genes amplified using qRT-PCR. Details of the primers using in qRT-PCR analysis. (DOCX 13 kb) [file 12885_2018_4227_MOESM2_ESM.docx]

| **Gene Name** | **Primer Sequences, 5′ to 3′** |
| --- | --- |
| *β-actin*  NM_001101 | **F:** CCTGGCACCCAGCACAAT  **R:** GCCGATCCACACGGAGTACT |
| *SRSF1*  NM_006924 | **F:** GATGGAATTGTGTTTTGCGTTTT **R:** CATCTACTCGTGCTGAATCCTT |
| *SRSF2*  NM_003016 | **F:** GTGCTTGGCTGTTTCCTGTTT **R:** CACTGTATGCTCCGTTATTTATATGC |
| *SRSF3*  NM_003017 | **F:** GTGAGAGAGTTGGTTGGTGTTG **R:** AAATGCGGCGGCTCAAATC |
| *SAM68*  NM_006559 | **F:** GCCACAGCCTCGGTCAAG **R:** AGTCTCCTTTCTGAATCTTCTCAATT |
| *HuR*  NM_001419 | **F**: TCGTCAACTACCTCCCTCAGA  **R:** GCTGTGTCCTGCTACTTTATCC |
| *HnRNP A1*  NM_002136 | **F:** CTTCATCCAGCCAAAGAGGTC  **R:** AAGTTGTCATTCCCACCGAAAC |
| *CLK1*  NM_004071 | **F:** TGAATACTATCTTGGGTTTACCGTAT  **R:** CGTTTCCTGGTTTTCTGTATCATAT |
| *SRPK1*  NM_003137 | **F:** TGGCCACAGGTGACTATTTG  **R:** CCCAAGGTTTCAGCTTCGT |
| *CLK1 (Fig.4)*  NM_004071 | **F:** GAACAAGCGCTGCAAATACA  **R**:CTGATACCGGCTTTCATGGT |
| *CLK3*  KJ896617.1 | **F**: TCTCAGGTTGCCCTGAAGAT  **R**:TAGGGGGTAAGGCTGGAAGT |
| *RPL13A*  KJ906094.1 | **F**:CCGGGTTGGCTGGAAAGGTAATTATG  **R**: CTTCTCGGCCTGTTTCCGTAC |
